# Extracellular electrical conductivity property imaging by decomposition of high-frequency conductivity at Larmor-frequency using multi-*b*-value diffusion-weighted imaging

**DOI:** 10.1371/journal.pone.0230903

**Published:** 2020-04-08

**Authors:** Mun Bae Lee, Geon-Ho Jahng, Hyung Joong Kim, Eung Je Woo, Oh In Kwon

**Affiliations:** 1 Department of Mathematics, Konkuk University, Seoul, Korea; 2 Department of Radiology, Kyung Hee University Hospital at Gangdong, College of Medicine, Kyung Hee University, Seoul, Korea; 3 Department of Biomedical Engineering, Kyung Hee University, Seoul, Korea; Henry Ford Health System, UNITED STATES

## Abstract

Magnetic resonance electrical properties tomography (MREPT) uses the B1 mapping technique to provide the high-frequency conductivity distribution at Larmor frequency that simultaneously reflects the intracellular and extracellular effects. In biological tissues, the electrical conductivity can be described as the concentration and mobility of charge carriers. For the water molecule diffusivity, diffusion weighted imaging (DWI) measures the random Brownian motion of water molecules within biological tissues. The DWI data can quantitatively access the mobility of microscopic water molecules within biological tissues. By measuring multi-*b*-value DWI data and the recovered high-frequency conductivity at Larmor frequency, we propose a new method to decompose the conductivity into the total ion concentration and mobility in the extracellular space (ECS) within a routinely applicable MR scan time. Using the measured multi-*b*-value DWI data, a constrained compartment model is designed to estimate the extracellular volume fraction and extracellular mean diffusivity. With the extracted extracellular volume fraction and water molecule diffusivity, we directly reconstruct the low-frequency electrical properties including the extracellular mean conductivity and extracellular conductivity tensor. To demonstrate the proposed method by comparing the ion concentration and the ion mobility, we conducted human experiments for the proposed low-frequency conductivity imaging. Human experiments verify that the proposed method can recover the low-frequency electrical properties using a conventional MRI scanner.

## Introduction

Using a magnetic resonance imaging (MRI) scanner, various techniques to measure and analyze the electrical properties of biological tissue have been developed and experimented [1–7]. The passive electrical properties of biological tissues appear complex phenomenon, depending on various factors, such as frequency, ion mobility, ion concentration, cell shapes, and cell membranes, etc. In biological tissues, frequency-dependent electrical properties such as permittivity and conductivity are divided into two electrically conducting compartments: the intracellular and extracellular spaces due to the cell membrane resistance, depending on frequency. The measurement of electrical conductivity of brain tissue has been initiated in the 1960s. Due to the insulation properties of thin cell membranes, internal electrical current flow caused by external current stimulation at low-frequency reflects ECS and cerebrospinal fluid (CSF), excluding the intracellular space (ICS). [1, 8, 9].

Magnetic resonance electrical impedance tomography (MREIT) has been developed to visualize electrical conductivity and/or current density images at low frequencies (below 1 kHz) by directly injecting low-frequency current through attached electrodes on the surface of imaging object [2, 4, 10, 11]. In MREIT, one component of the induced magnetic flux density is acquired using an MRI scanner. Most MREIT algorithms have focused on visualizing the isotropic conductivity distribution by using only one component of the magnetic flux density data [12–14]. Combining the diffusion tensor imaging (DTI) and MREIT techniques, DT-MREIT method, which is a direct method for absolute conductivity tensor image, was proposed based on the linear relationship between the water diffusion tensor and the electrical conductivity tensor [3, 15]. MREIT technique is a direct method to investigate the internal low-frequency electrical properties, however, various difficulties still need to be overcome due to the external direct current injection into the human body.

For the high-frequency conductivity using a conventional MRI scanner without any external electrical stimulation, magnetic resonance electrical properties tomography (MREPT) techniques successfully recover the conductivity distribution at Larmor frequency (about 128 MHz at 3 T) [7, 16, 17]. Since the electrical conductivity of biological tissues is primarily determined by the concentration and mobility of ions, the electrical conductivity can be sensitive to the changes of physiological and pathological conditions of tissues and organs [16]. A recent work using MREPT technique shows that the recovered high-frequency electrical conductivity is sensitive to microstructural changes of tissues due to irradiation [18].

Using the B1 mapping technique, the high-frequency electric conductivity at Larmor frequency, which simultaneously reflects the combined electrical properties in ICS and ECS, can be formally decomposed into a two compartment conductivity model specific to ICS and ECS. In each compartment, the electrical conductivity can be expressed as the product of concentration and mobility of charge carriers such as ions and charged molecules. In the case of biological tissue containing the intracellular and extracellular fluids, the extracellular matrix material and the cells with insulating membrane also contribute to the conductivity of biological tissues [19].

Diffusion-weighted imaging (DWI), based on the diffusion of water molecules in tissues, provides potentially information on the brain functional activity, *in vivo* and non-invasively. Multi-*b*-value DWIs can probe the microstructure of neural tissues and analyze its hindrance to water diffusion using various signal models [20–24]. Intravoxel incoherent motion (IVIM) imaging is a model for evaluating the diffusion procedure in biological tissues using a bi-exponential model [20, 21]. The bi-exponential model is based on the assumption that the slow diffusion is attributed to water molecules interacting with the cell membranes and associated cell structures, while the fast diffusion component occur in less restricted water environments found in both intracellular and extracellular spaces. Both slow and fast water pools may coexist in the intracellular compartment.

Lately, an electrodeless method providing the low-frequency conductivity tensor image (LF-CTI) using a clinical MRI scanner without any external hardware has been initiated [25, 26]. The proposed LF-CTI method based on the observation that the conductivity is proportional to the product of mobility and concentration of charge carriers. Since MREPT technique can recover the high-frequency conductivity at Larmor-frequency, the LF-CTI method mainly focuses on separating the ion mobility and concentration in ECS from the recovered high-frequency conductivity.

The LF-CTI method provides a possible way to visualize the low-frequency conductivity tensor map without additional external injection current, human imaging experiments, however, were deferred mainly due to lack of validation studies. The LF-CTI method needs to overcome several difficulties [25, 26]:

- To separate the recovered high-frequency conductivity into the intracellular and extracellular compartments, the LF-CTI method solved a three compartment model to estimate the extracellular volume fraction and diffusion coefficients in ECS and ICS. The determination of diffusion coefficients and extracellular volume fraction from the three pool model, that is a combination of multi-exponential curves, is highly sensitive to the measured noise.
- DWI signals reflected the effects of ICS and ECS, but the LF-CTI method used the diffusion tensor at *b* = 800 s/mm^2^ as the extracellular diffusion tensor.
- The LF-CTI method requires a relatively long MR scan time due to the number of diffusion gradients and multi-*b*-value DWIs.

We propose a method to provide a low-frequency electrical conductivity property imaging using B1 phase map and multi-*b*-value DWI data (M*b*D-LF-CPI). The proposed M*b*D-LF-CPI method separates the apparent extracellular ion concentration and mobility, acquired within clinically applicable MR scan time. With an additional diffusion tensor at a fixed *b*-value, a method for the low-frequency conductivity tensor imaging is also proposed.

We introduce a new multi-compartment model to stably extract the extracellular mean diffusivity, extracellular volume fraction, and isotropic volume fraction, which are parameters related to multi-*b*-value DWIs. The proposed method is motivated by the neurite orientation dispersion and density imaging (NODDI), a widely used model to infer microstructural features in the brain [23]. NODDI uses three types of microstructural environment (intra-neurite, extra-neurite, and CSF) to estimate a neurite density index (NDI) and an orientation dispersion index (ODI). NODDI requires multiple *b*-values and many diffusion gradient directions. The anisotropic intra-neurite compartment was modeled as a set of sticks, assuming unhindered diffusion along the neurite to capture the highly restricted diffusion properties [27, 28].

The relatively long acquisition time for NODDI characterizing the dispersion and density has been the limitation for clinical applications due to high scan costs. To separate low-frequency conductivity from the high-frequency conductivity, the proposed method only needs the extracellular volume fraction and diffusion coefficient, not the orientation dispersion recovered from a conventional NODDI. To characterize the total ion concentration weighted part in the brain region, we reasonably assume that the mobility of charge carriers is proportional to that of water molecules in the same structural environment. The proposed model includes three unknowns to be determined such as extracellular volume fraction, extracellular mean diffusivity, and isotropic volume fraction. With the high-frequency conductivity and the estimated parameters using the proposed model, we reconstruct the low-frequency mean conductivity.

After the determination of low-frequency mean conductivity, using the estimated extracellular diffusivity and the measured water diffusion tensor at a fixed *b*-value, to extract the low-frequency anisotropic electrical properties, we propose a method of separating the diffusion tensor at a fixed *b*-value into the intracellular and extracellular diffusion tensors.

To demonstrate the proposed method, we conducted human experiments and recovered the high-frequency conductivity using the measured B1 phase map. For MREPT experiments, a multi-spin-echo pulse sequence with multiple refocusing pulses was used to reduce measured noise artifact. Since measured multi-*b*-value DWIs exponentially decayed, it is difficult to avoid the noise amplification from the three compartment model even with 15 *b*-values [25]. To quantitatively compare the quality of reconstructed parameters, we used 7 and 4 *b*-value DWIs to extract the microstructural parameters such as the volume fraction and mean diffusivity in ECS. The extracellular diffusion tensor map was recovered through the estimated extracellular diffusivity and the measured diffusion tensor map at a fixed *b*-value. The low-frequency conductivity tensor was recovered using the extracellular diffusion tensor map, the microstructural parameters with multi-*b*-value DWIs, and high-frequency conductivity. The human experiments verified that the proposed method has the potential to rapidly recover the low-frequency electrical properties without any additional external injection current.

## Materials and methods

### Preliminary

#### High-frequency conductivity using B1-map

The high-frequency electrical tissue properties of conductivity *σ*_*H*_ and permittivity *ϵ*_*H*_ satisfy the following at Larmor frequency *ω*

$$\begin{array}{c} \nabla^{2}B_{1}=i\omega \mu_{0}\gamma_{H}B_{1}-\frac{\nabla \gamma_{H}}{\gamma_{H}}\times \left(\nabla \times B_{1}\right) \end{array}$$

where *γ*_*H*_ = *σ*_*H*_ + *iωϵ*_*H*_, **B**_1_ denotes the B1 field and *μ*_0_ = 4*π* × 10^−7^ N/A^2^ is the magnetic permeability of free space [7]. For the positive (negative) rotating component of the transmit B1 field $B_{1}^{+}=|\;B_{1}^{+}\;|e^{i\phi^{+}}$ ($B_{1}^{-}=|\;B_{1}^{-}\;|e^{i\phi^{-}}$), by assuming *σ*_*H*_ ≫ *ωϵ*_*H*_, a phase-based convection reaction equation-based MREPT formula was derived as

$$\begin{array}{c} (\nabla \phi^{tr}\cdot \nabla (\frac{1}{\sigma_{H}}))+\frac{\nabla^{2}\phi^{tr}}{\sigma_{H}}-2\omega \mu_{0}=0 \end{array}$$ (1)

where *ϕ*^*tr*^ = *ϕ*^+^ + *ϕ*^−^ is the transceiver phase using MRI [6]. To stabilize the formula (1), after adding an artificial diffusion term, the Eq (1) leads to

$$\begin{array}{c} -c\nabla^{2}(\frac{1}{\sigma_{H}})+(\nabla \phi^{tr}\cdot \nabla (\frac{1}{\sigma_{H}}))+\frac{\nabla^{2}\phi^{tr}}{\sigma_{H}}=2\omega \mu_{0} \end{array}$$ (2)

where *c* is a constant diffusion coefficient.

#### Multi-*b*-value DWI

In DWI, two magnetic field gradients with the same area between 180° RF pulse are used. The first gradient induces dephasing of water proton spins and the second gradient refocuses the spins. The amount of diffusion weighting can be controlled by modifying the *b*-value, which is related with the gradient area, strength, and time spacing between the two magnetic filed gradients. The signal intensity *S*_*j*_ by applying a diffusion encoding gradient is given by

$$\begin{array}{c} S_{j}=S_{0}\exp\left(-b_{j}D\right),\;j=1,\cdots ,N_{b} \end{array}$$

where *S*_0_ is the signal obtained without diffusion gradient and *b*_*j*_ denotes the diffusion-weighting factor depending on the gradient pulse used in the DWI sequence:

$$\begin{array}{c} b_{j}=\gamma^{2}\delta_{j}^{2}G_{j}^{2}(\Delta_{j}-\frac{\delta_{j}}{3}) \end{array}$$

where *γ* = 26.75 × 10^7^ *rad*/*Ts* is the gyromagnetic ratio of hydrogen, Δ_*j*_ is the diffusion time interval, and *δ*_*j*_ and *G*_*j*_ are the duration and amplitude, respectively, of the diffusion-sensitizing gradient pulse along a given direction.

#### Decomposition of high-frequency conductivity *σ*_*H*_

Biological tissues contain several charge carriers. K^+^, Na^+^, Ca^2+^, Cl^−^, and several ions are dominant charge carriers in the extracellular and intracellular fluids. Using the Einstein relation between the diffusivity and mobility of charge carriers, the electrical mobility *m*_*j*_ of the *j*-th charge carrier in biological tissues can be expressed as

$$\begin{array}{c} m_{j}=(\frac{q}{k_{B}T})d_{j}=(\frac{r_{w}q}{r_{j}k_{B}T})d_{w} \end{array}$$

where *q* = 1.6 × 10^−19^C is the absolute value of the charge of electron, *k*_*B*_ is the Boltzmann constant, *r*_*w*_ and *r*_*j*_ are the Stokes radius of a water molecule and an ion, respectively, *d*_*w*_ is the diffusion coefficient of a water molecule, and *T* is the absolute temperature.

The diffusion coefficient *d*_*w*_ is related to the medium viscosity and Stokes radius of the water molecule. Using the relation between the apparent conductivity *σ*_*a*_ and diffusion coefficient, the apparent electrical conductivity in the biological tissues can be represented as

$$\begin{array}{c} \sigma_{a}=\sum_{j=1}^{N_{E}}c_{j}m_{j}=\sum_{j=1}^{N_{E}}c_{j}(\frac{r_{w}q}{r_{j}k_{B}T})d_{w}={\bar{c}}_{a}d_{w} \end{array}$$

where *c*_*j*_ denotes the concentration of the *j*-th ion. In this paper, we set ${\bar{c}}_{a}=\sum_{j=1}^{N_{E}}c_{j}(\frac{r_{w}q}{r_{j}k_{B}T})$ as the apparent total ion-concentration for the water molecule.

The reconstructed high-frequency conductivity can be decomposed into the extracellular and intracellular compartments:

$$\begin{array}{cc} \sigma_{H} & =\sigma_{L}+\sigma_{I}\\ & =\alpha {\bar{c}}_{e}d_{w}^{e}+\left(1-\alpha \right){\bar{c}}_{i}d_{w}^{i} \end{array}$$ (3)

where *α* denotes the extracellular volume fraction in a voxel and ${\bar{c}}_{e}=\sum_{j=1}^{N_{e}}c_{j}^{e}(\frac{r_{w}q}{r_{j}k_{B}T})$, ${\bar{c}}_{i}=\sum_{j=1}^{N_{i}}c_{j}^{i}(\frac{r_{w}q}{r_{j}k_{B}T})$, and $d_{w}^{e}$ and $d_{w}^{i}$ are the water diffusion coefficients in ECS and ICS, respectively. Here, the low-frequency conductivity $\sigma_{L}=\alpha {\bar{c}}_{e}d_{w}^{e}$ denotes the extracellular conductivity.

### Microstructural parameters using multi-*b*-value DWIs

To investigate the microstructure of biological tissues using the diffusion related parameters obtained from multi-*b*-value DWIs, the most common method is DTI, which describes diffusion coefficients by a 3 × 3 symmetric matrix. The bi-tensor model has been solved by increasing the number of diffusion gradients and multi-*b*-value DWIs, at the cost of long scan time [29]. Multi-compartment models are typically used to eliminate CSF contamination in NODDI [23]. Intravoxel incoherent motion (IVIM) is also a method using multi-*b*-value DWIs to extract information about the microcirculation and microvasculature in addition to the diffusion parameters:

$$\begin{array}{c} S_{j}=S_{0}\left(f_{b}\;\text{exp}\;\left(-b_{j}\left(D_{1}+D^{*}\right)\right)+\left(1-f_{b}\right)\;\text{exp}\;\left(-b_{j}D_{2}\right)\right) \end{array}$$ (4)

where *f*_*b*_ is the perfusion fraction, D* is the psedo-diffusion coefficient, *D*_1_ is the water diffusion coefficient in blood, and *D*_2_ is the water diffusion coefficient in the tissue [21]. The pseudo-diffusion coefficient D* describes the incoherent motion of blood within the capillary network. Since the perfusion fraction *f*_*b*_ includes the intracellular diffusivity component, the two-compartment model in (4) is not appropriate to extract the low-frequency conductivity component from the recovered high-frequency conductivity *σ*_*H*_ in (3).

NODDI estimates the neurite orientation and dispersion parameters by distinguishing the brain tissue with three types of microstructural compartments: CSF, anisotropic hindered diffusion (extra-neurite space), and anisotropic restricted diffusion (intra-neurite space) [23]. NODDI uses the following three compartment model:

$$\begin{array}{c} A=\left(1-\nu_{iso}\right)\left(\nu_{ic}A_{ic}+\left(1-\nu_{ic}\right)A_{ec}\right)+\nu_{iso}A_{iso} \end{array}$$ (5)

where *A*_*ic*_ and *ν*_*ic*_ are the normalized signal from water restricted by a directionally orientated cylinder and intra-neurite volume fraction, respectively; *A*_*ec*_ is the normalized signal of the extra-neurite compartment and *A*_*iso*_ and *ν*_*iso*_ are the normalized signal and the volume fraction of the CSF compartment, respectively. *A*_*ic*_ is represented as

$$\begin{array}{c} A_{ic}=\int_{S^{2}}\rho \left(\vec{n}\right)e^{-bd_{∥}{\left(\vec{q}\cdot \vec{n}\right)}^{2}}\;d\vec{n} \end{array}$$ (6)

where *ρ* is the spherical function that is non-negative, antipodal symmetry, and integrates to 1 over the unit sphere, $\vec{q}$ is the gradient direction, and $\vec{n}$ is an outward unit vector. $e^{-bd_{∥}{\left(\vec{q}\cdot \vec{n}\right)}^{2}}$ denotes the signal from water restricted by a stick with diffusivity *d*_∥_ and orientation $\vec{n}$ [23]. To estimate the orientation dispersion index, which is a component of $\rho (\vec{n})$, NODDI requires relatively many gradient directions to cover the unit sphere *S*^2^.

The anisotropic intra-neurite compartment is modeled as a set of sticks to capture the highly restricted diffusion property, which is unhindered diffusion along neurites. The extra-neurite compartment refers to the space hindered by the presence of neurites. The CSF compartment is modeled as isotropic Gaussian diffusion with diffusivity *d*_*iso*_. NODDI uses *a priori* model parameters to stabilize the reconstruction procedure; intrinsic free diffusivity for the intra-neurite *d*_∥_(= *d*_*ic*_) = 1.7 × 10^−3^mm^2^/s and *d*_*iso*_ = 3.0 × 10^−3^mm^2^/s [23].

The NODDI has a sufficient potential as a practical medical image, but still has a problem in clinical applications due to high scan costs. It is important to reduce a number of DWIs to fill the *q*-space with different diffusion weights and gradient directions. From the point of view of electrical conductivity, a goal is to separate low-frequency conductivity component, $\sigma_{L}=\alpha {\bar{c}}_{e}d_{w}^{e}$ in (3), from the high-frequency conductivity *σ*_*H*_. By modifying the NODDI model to only extract the volume fractions *ν*_*ic*_ and *ν*_*iso*_ in (5), and the extracellular mean diffusivity, to extract the low-frequency conductivity component, we propose a multi-compartment model only depending on multi-*b*-value DWIs:

$$\begin{array}{cc} {\hat{S}}_{j}/S_{0} & =(1-\nu_{iso})(\nu_{ic}\;\text{exp}\;\left(-b_{j}\nu_{ic}d_{ic}\right)\\ & +\left(1-\nu_{ic}\right)\;\text{exp}\;\left(-b_{j}\left(1-\nu_{ic}\right)d_{*}^{e}\right))\\ & +\nu_{iso}\;\text{exp}\;\left(-b_{j}d_{iso}\right)+\nu_{0},\;j=1,\cdots ,N_{b} \end{array}$$ (7)

where *d*_*ic*_ = 1.7 × 10^−3^mm^2^/s and *d*_*iso*_ = 3.0 × 10^−3^mm^2^/s. The proposed model includes four unknowns: intracellular volume fraction *ν*_*ic*_, extracellular diffusivity $\left(1-\nu_{ic}\right)d_{*}^{e}$, isotropic volume fraction *ν*_*iso*_, and offset value *ν*_0_. By solving the following nonlinear least square problem, we can determine the parameters to separate the intracellular and extracellular compartments:

$$\begin{array}{c} \min_{\nu_{ic},d_{*}^{e},\nu_{iso},\nu_{0}}∥\;\vec{S}-\vec{\hat{S}}\left(\nu_{ic},d_{*}^{e},\nu_{iso},\nu_{0}\right)\;∥ \end{array}$$

where $\vec{S}=\left(S_{1},\cdots ,S_{N_{b}}\right)$ is the measured DWI signals for multi-*b*-value DWIs and $\vec{\hat{S}}\left(\nu_{ic},d_{*}^{e},\nu_{iso},\nu_{0}\right)=\left({\hat{S}}_{1},\cdots ,{\hat{S}}_{N_{b}}\right)$ denotes the generated DWI signals by using the proposed compartment model (7).

The extracellular volume fraction *α* in (3) is estimated at each voxel as

$$\begin{array}{c} \alpha =\left(1-\nu_{iso}\right)\left(1-\nu_{ic}\right)+\nu_{iso} \end{array}$$ (8)

Using the relation, $\alpha d_{w}^{e}=\left(1-\nu_{iso}\right)\left(1-\nu_{ic}\right)\left(1-\nu_{i}\right)d_{*}^{e}+\nu_{iso}d_{iso}$, the extracellular mean diffusivity can be estimated as

$$\begin{array}{c} d_{w}^{e}=\frac{\left(1-\nu_{iso}\right){\left(1-\nu_{ic}\right)}^{2}d_{*}^{e}}{\left(1-\nu_{iso}\right)\left(1-\nu_{ic}\right)+\nu_{iso}}+\frac{\nu_{iso}d_{iso}}{\left(1-\nu_{iso}\right)\left(1-\nu_{ic}\right)+\nu_{iso}} \end{array}$$

The intracellular mean diffusivity is expressed as

$$\begin{array}{c} d_{w}^{i}=\nu_{ic}d_{ic} \end{array}$$

### Low-frequency mean conductivity

To estimate the ratio of ion concentrations in ICS and ECS, for the human brain, the ratio value $\beta =\frac{{\bar{c}}_{i}}{{\bar{c}}_{e}}=0.41$ was suggested by adopting reference values of intracellular and extracellular ion concentrations of four predominant ions (Na^+^, Cl^−^, K^+^, and Ca^2+^) [25, 26]. Using the reference ratio value *β* = 0.41, the apparent extracellular ion concentration ${\bar{c}}_{e}$ can be estimated as

$$\begin{array}{c} {\bar{c}}_{e}=\frac{\sigma_{H}}{\alpha d_{w}^{e}+\left(1-\alpha \right)\beta d_{w}^{i}} \end{array}$$ (9)

By subtracting the intracellular compartment of *σ*_*H*_, the low-frequency mean conductivity, *σ*_*L*_, is expressed as

$$\begin{array}{c} \sigma_{L}=\alpha \frac{\sigma_{H}}{\alpha d_{w}^{e}+\left(1-\alpha \right)\beta d_{w}^{i}}d_{w}^{e} \end{array}$$

### Low-frequency anisotropic conductivity tensor using the relationship between extracellular mean diffusivity and extracellular diffusion tensor

The measured diffusion tensor **D**_*b*_ at a fixed *b*-value can be written as a positive definite symmetric matrix:

$$\begin{array}{c} D_{b}=S_{D}{\tilde{D}}_{b}S_{D}^{T}\;\text{with}\;{\tilde{D}}_{b}=(\begin{array}{ccc} d_{1}^{b} & 0 & 0\\ 0 & d_{2}^{b} & 0\\ 0 & 0 & d_{3}^{b} \end{array}) \end{array}$$

where the column vectors of $S_{D}$ are the orthonormal eigenvectors of **D**_*b*_, the superscript *T* denotes the transpose and $d_{1}^{b}\leq d_{2}^{b}\leq d_{3}^{b}$ are the corresponding eigenvalues.

We separate the apparent diffusion tensor **D**_*b*_ into the extracellular and intracellular compartments:

$$\begin{array}{c} D_{b}=\alpha D_{ext}+\left(1-\alpha \right)D_{int} \end{array}$$

where **D**_*ext*_ and **D**_*int*_ denote the apparent diffusion tensors in ECS and ICS, respectively. By assuming that the diffusion tensors **D**_*ext*_ and **D**_*b*_ share the eigenvectors, the extracellular diffusion tensor **D**_*ext*_ can be expressed as

$$\begin{array}{c} D_{ext}=S_{D}{\tilde{D}}_{ext}S_{D}^{T}\;\text{with}\;{\tilde{D}}_{ext}=(\begin{array}{ccc} d_{1}^{ext} & 0 & 0\\ 0 & d_{2}^{ext} & 0\\ 0 & 0 & d_{3}^{ext} \end{array}) \end{array}$$ (10)

Using the estimated extracellular mean diffusivity $d_{w}^{e}$ and the relation $d_{w}^{e}=\left(d_{1}^{ext}+d_{2}^{ext}+d_{3}^{ext}\right)/3$, we define a scale parameter *η* as

$$\begin{array}{c} \eta =\frac{3d_{w}^{e}}{d_{1}^{b}+d_{2}^{b}+d_{3}^{b}} \end{array}$$ (11)

Under the hypothesis that the extracellular diffusion tensor **D**_*ext*_ and the diffusion tensor **D**_*b*_ share the eigenvectors, we can determine the extracellular diffusion tensor:

$$\begin{array}{c} D_{ext}=\eta D_{b} \end{array}$$ (12)

From the relation (12), the low-frequency conductivity tensor can be expressed as the following

$$\begin{array}{c} C_{L}=\alpha {\bar{c}}_{e}D_{ext}=\alpha \eta {\bar{c}}_{e}D_{b} \end{array}$$ (13)

where the ion mobility is assumed to be proportional to the water molecule diffusion flow.

### Human experiments setup

Two healthy volunteers without a documented history of any disease were recruited. The participants were located inside the bore of a 3T MRI scanner with a 32-channel RF head coil (Achieva TX, Philips Medical Systems, the Netherlands). All experimental protocols were approved by the institutional review board of Kyung Hee University (KHSIRB-16-033). All methods were carried out in accordance with the relevant guidelines and regulations and all participants provided written informed consent.

For MREPT imaging experiments, the multi-spin-echo pulse sequence with multiple refocusing pulses was adopted. Before the data acquisition, we applied a volume shimming method with the volume defined to cover the brain region. Imaging parameters were as follows: repetition time *T*_*R*_ = 1500 ms, echo time *T*_*E*_ = 15 ms, number of echoes (NE) = 6, number of excitation (NEX) = 1, slice thickness = 4 mm, number of slices = 5, acquisition matrix = 128×128, field-of-view (FOV) = 240 × ?240 mm^2^, and scan time = 16 min.

DWI data sets were obtained using the single-shot spin-echo echo planner imaging (SS-SE-EPI) pulse sequence [30]. We applied the diffusion weighting gradients in 15 directions with 15 *b*-values of 50, 150, 300, 500, 700, 1000, 1400, 1800, 2200, 2600, 3000, 3600, 4000, 4500, and 5000 s/mm^2^, respectively. Imaging parameters were as follows: *T*_*R*_ = 2000 ms, *T*_*E*_ = 70 ms, flip angle = 90°, NEX = 2, slice thickness = 4 mm, number of slices = 5, acquisition matrix = 64 × 64 and scan time = 23 min. The matrix size of 64 × 64 was extended to 128 × 128 to match the spatial resolution of MREPT experiment. We used only 3 orthogonal gradient directions in the experiments and the results were also compared to the results estimated using 15 gradient directions.

**Fig 1:**
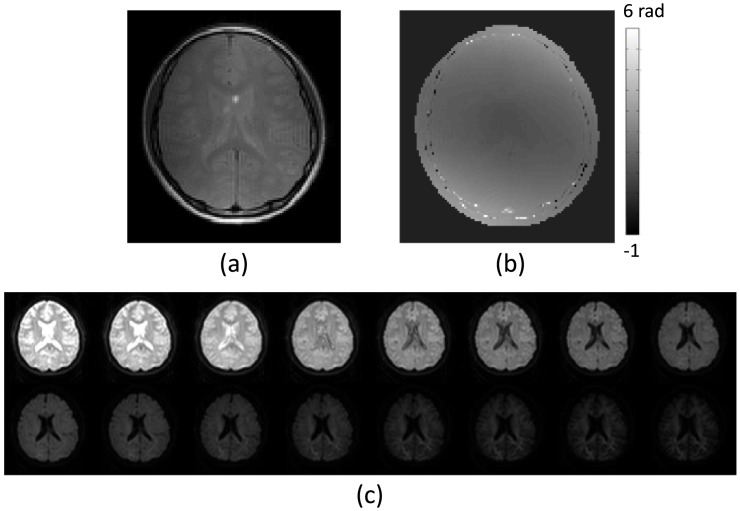
MR magnitude and B1 phase images. (a) MR magnitude image and (b) B1 phase image at the first imaging slice using the spin MR pulse sequence. (c) MR magnitude images corresponding to multi-*b*-value: *b* = 0, 50, 150, 300, 500, 700, 1000, 1400, 1800, 2200, 2600, 3000, 3600, 4000, 4500, and 5000 s/mm^2^, respectively.

Fig 1(a) and 1(b) show the MR magnitude and the B1 phase image at the first imaging slice using the spin MR pulse sequence, respectively. Fig 1(c) shows MR magnitude variations corresponding to multi-*b*-values: *b* = 0, 50, 150, 300, 500, 700, 1000, 1400, 1800, 2200, 2600, 3000, 3600, 4000, 4500, and 5000 s/mm^2^, respectively.

## Results

### Low-frequency mean conductivity

We reconstructed the high-frequency conductivity, *σ*_*H*_, with the acquired transceiver phases of the B1 maps (Fig 1(b)) by solving the partial differential equation in (3).

To avoid the background phase signal due to the consecutive 180° RF pulses, we used odd echoes of six measured complex signals to reduce measured noise. Since the amount of noise in the phase signal is inversely proportional to MR magnitude intensity, ${\tilde{S}}_{k},\;k=1,3,5$, the measured phase signal was optimized as a weighted averaging using the weight of [31]

$$\begin{array}{c} w_{k}=\frac{|{\tilde{S}}_{k}{|}^{2}}{|{\tilde{S}}_{1}{|}^{2}+|{\tilde{S}}_{2}{|}^{2}+{\left|{\tilde{S}}_{3}\right|}^{2}},\;k=1,3,5 \end{array}$$

**Fig 2:**
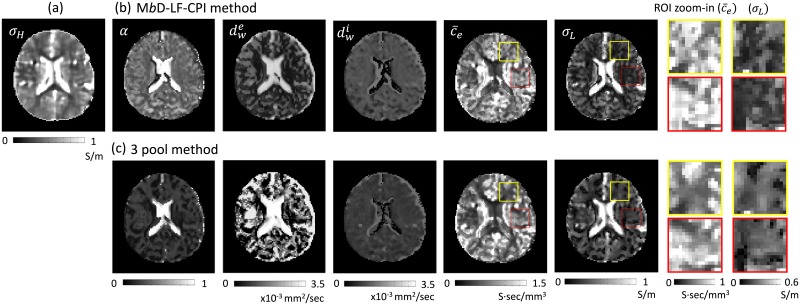
Comparisons of the results using M*b*D-LF-CPI and the three pool method in (14). (a) Recovered high-frequency conductivity *σ*_*H*_. (b) Recovered extracellular volume fraction *α*, extracellular mean diffusivity $d_{w}^{e}$, intracellular diffusivity $d_{w}^{i}$, apparent extracellular ion concentration ${\bar{c}}_{e}$ and low-frequency mean conductivity *σ*_*L*_ images, respectively, using M*b*D-LF-CPI method. (c) Recovered results corresponding to the results in (b) using the three pool model method in (14). To show the difference more clearly, the last two columns zoom in on the area marked by the rectangles.

Fig 2(a) shows the recovered high-frequency conductivity image in the first imaging slice of the brain. To solve the Eq (2), we discretized using three-point central difference approximation to derive a matrix system **Ax** = **b** in the brain region. To stabilize the reaction-diffusion Eq (2), we used the diffusion term *c* = 0.025 to estimate the high-frequency conductivity images. The estimated conductivity values were slightly lower than the known reference conductivity values because the evaluation procedure for the conductivity included the numerical differentiations and the regularization parameter *c* in (2).

Fig 2(b) shows the extracellular volume fraction *α*, extracellular mean diffusivity $d_{w}^{e}$, intracellular diffusivity $d_{w}^{i}$, apparent extracellular ion concentration ${\bar{c}}_{e}$ and low-frequency mean conductivity *σ*_*L*_, respectively, using the proposed M*b*D-LF-CPI method.

We designed the diffusion coefficient term in the intracellular compartment by including the intracellular volume fraction information, exp(−*b*_*j*_ *ν*_*ic*_ *d*_*ic*_), in (7). Using the high-frequency conductivity and the estimate diffusion parameters, we recovered an apparent extracellular ion concentration, ${\bar{c}}_{e}$, in (9). The extracellular ion concentration in the white matter region was lower than in other regions, especially in the genu and splenium of the corpus where the density of thin unmyelinated fibers was high. Comparing to the high-frequency conductivity *σ*_*H*_, the low-frequency mean conductivity *σ*_*L*_ shows different electrical characteristics in Fig 2(b).

Since the given observable multi-*b*-value DWI data are a combination of exponentially decay signals depending on the diffusion gradient strength, the method of estimating the extracellular volume fraction, intracellular diffusivity and extracellular mean diffusivity is sensitive to the measured noise. Due to the ill-posedness, the first proposed electrodeless method fitted the exponentially decay signals by using the three pool model [25]:

$$\begin{array}{cc} {\hat{S}}_{j}/S_{0} & =\nu_{ecm}\;\text{exp}\;\left(-b_{j}d_{ecm}^{w}\right)+\nu_{ecm}\;\text{exp}\;\left(-b_{j}d_{ecw}^{w}\right)\\ & +\nu_{i}\;\text{exp}\;\left(-b_{j}d_{i}^{w}\right)+\nu_{0},\;j=1,\cdots ,N_{b} \end{array}$$ (14)

where $d_{ecw}^{w}=3.0\times 10^{-3}{\text{mm}}^{2}/\text{s}$.

Using the three pool model in (14), the extracellular volume fraction *α* and extracellular diffusion coefficient $d_{w}^{e}$ are estimated at each voxel as

$$\begin{array}{c} \alpha =\frac{\nu_{ecm}+\nu_{ecw}}{\nu_{ecm}+\nu_{ecw}+\nu_{i}} \end{array}$$

and

$$\begin{array}{c} d_{w}^{e}=\frac{\nu_{ecm}}{\nu_{ecm}+\nu_{ecw}}d_{ecm}^{w}+\frac{\nu_{ecw}}{\nu_{ecm}+\nu_{ecw}}d_{ecw}^{w}. \end{array}$$

In this paper, we compared the reconstructed results using the proposed method to those using the three pool model in (14): Fig 2(c) shows the results of the three pool method. To show the difference more clearly, the last two columns zoom in on the area marked by the rectangles.

Experiment results for the other human subject are included in the supporting information (S1 Fig).

### Low-frequency conductivity tensor

**Fig 3:**
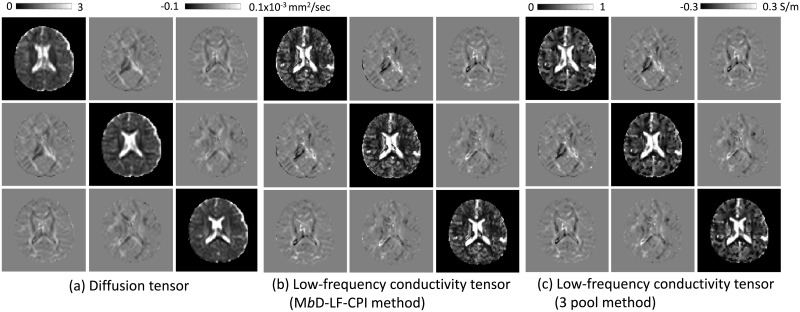
Diffusion and conductivity tensors. (a) Water molecule diffusion tensor using the *b* value of 1000 s/mm^2^ in the first slice. (b) Reconstructed low-frequency conductivity tensor images using the proposed M*b*D-LF-CPI method. (c) Reconstructed low-frequency conductivity tensor images using the three pool model in (14).

Fig 3(a) shows the 3 × 3 diffusion tensor components using DWIs with *b*-value of 1000 s/mm^2^. To extract the water molecule diffusion tensor in ECS, we solved the Eqs (11) and (12) using the estimated extracellular mean diffusivity $d_{w}^{e}$ and the diffusion tensor **D**_*b*_. From the estimated extracellular diffusion tensor **D**_*ext*_, the extracellular volume fraction *α* = (1 − *ν*_*iso*_)(1 − *ν*_*ic*_) + *ν*_*iso*_ in (8), and the extracellular ion concentration ${\bar{c}}_{e}=\frac{\sigma_{H}}{\alpha d_{w}^{e}+\left(1-\alpha \right)\beta d_{w}^{i}}$ in (9), we reconstructed the low-frequency conductivity tensor map **C**_*L*_ in (13), which was displayed in Fig 3(b).

The estimated low-frequency conductivity tensor using the proposed method was slightly low in the white matter region. Fig 3(c) shows the estimated low-frequency conductivity tensor map corresponding to the three pool method. S2 Fig shows the corresponding results in Fig 3.

To verify the proposed method, the brain region was segmented into CSF, gray matter (GM), and white matter (WM) regions. Table 1(a) shows the estimated high-frequency conductivity, apparent extracellular ion concentration, low-frequency mean conductivity, and the diagonal components of the reconstructed low-frequency conductivity tensor in the segmented ROIs. The conductivity of WM, composed of myelinated nerve axons, is highly anisotropic with different longitudinal and transverse conductivity. Especially, in WM region, the low-frequency conductivity seems to be more hindered. The low-frequency conductivity, estimated in WM region, was relatively smaller than the high-frequency conductivity.

**Table 1:** Results of the proposed M*b*D-LF-CPI method and the three pool method. Estimated high-frequency conductivity *σ*_*H*_, extracellular ion concentration ${\bar{c}}_{e}$, low-frequency mean conductivity *σ*_*L*_, diagonal components of reconstructed low-frequency conductivity tensor **C**_*L*_ measured within the ROIs. The numbers of pixels in the ROIs are shown in the last columns.

(a) M*b*D-LF-CPI method							
	*σ*_*H*_	${\bar{c}}_{e}$	*σ*_*L*_	*C*_11_	*C*_22_	*C*_33_	Pixels
CSF	1.19±0.40	0.45 ±0.28	1.18 ±0.40	1.09 ±0.39	1.25 ±0.46	1.19 ±0.44	386
GM	0.55±0.17	0.93 ±0.46	0.33 ±0.21	0.34 ±0.22	0.34 ±0.21	0.32 ±0.22	1576
WM	0.41±0.13	0.87 ±0.40	0.19 ±0.11	0.20 ±0.12	0.18 ±0.11	0.20 ±0.13	2191
(b) three pool method							
	*σ*_*H*_	${\bar{c}}_{e}$	*σ*_*L*_	*C*_11_	*C*_22_	*C*_33_	Pixels
CSF	1.19±0.40	0.44 ±0.28	1.18 ±0.40	1.10 ±0.39	1.26 ±0.46	1.20 ±0.45	386
GM	0.55±0.17	0.90 ±0.47	0.34 ±0.24	0.34 ±0.24	0.35 ±0.24	0.32 ±0.24	1576
WM	0.41±0.13	0.76 ±0.45	0.21 ±0.11	0.21 ±0.12	0.19 ±0.11	0.21 ±0.14	2191

The diagonal components of the diffusion tensor in CSF region were 2.5±0.6 × 10^−3^, 2.9±0.7 × 10^−3^, and 2.8±0.8 × 10^−3^ mm^2^/s, respectively. Thus, in CSF region, the second component of the recovered low-frequency conductivity tensor *C*_22_ was relatively high. The mean of diagonal components of the low-frequency conductivity tensor in CSF was 1.18±0.43 S/m, which was similar to the high-frequency conductivity value 1.19±0.40 S/m in CSF.

Table 1(b) summarizes the results of the three pool method (See also S1 Table).

**Fig 4:**
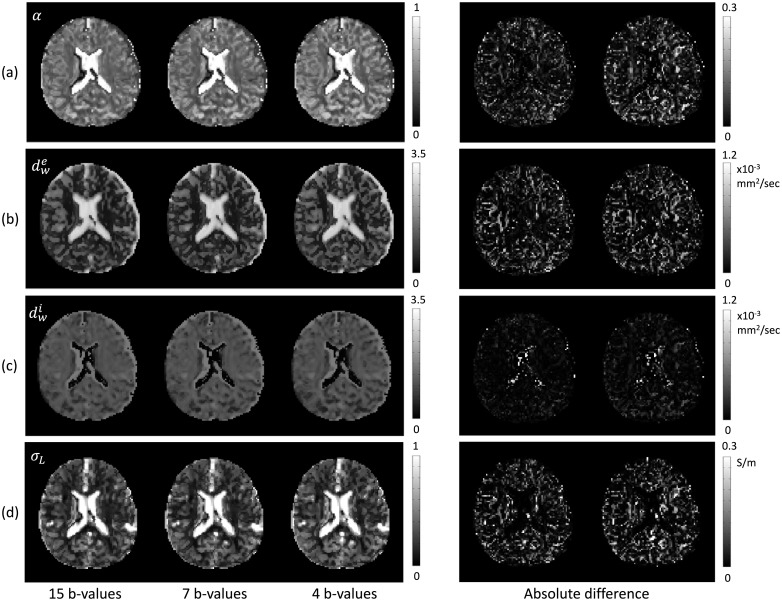
Results of the proposed M*b*D-LF-CPI method. Comparison of reconstructed results estimated with 15, 7, and 4 *b*-value DWIs in the first imaging slice of the brain. (a) extracellular volume fraction *α*, (b) extracellular mean diffusivity $d_{w}^{e}$, (c) intracellular diffusivity $d_{w}^{i}$, and (d) low-frequency mean conductivity *σ*_*L*_. The 4-th and 5-th columns show the absolute values of difference between the results using 15 *b*-value DWIs with the results using 7 and 4 *b*-value DWIs, respectively.

**Fig 5:**
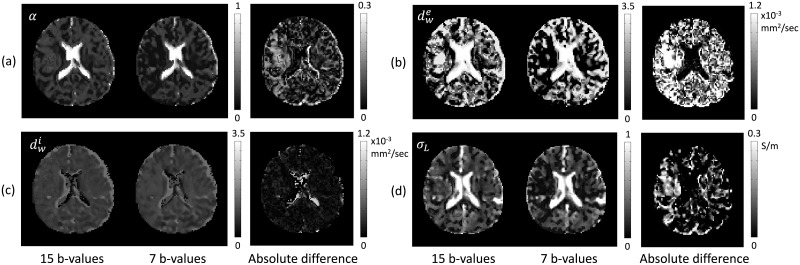
Results of the three pool method. Comparison of reconstructed results estimated with 15 and 7 *b*-value DWIs in the first imaging slice of the brain. (a) extracellular volume fraction *α*, (b) extracellular mean diffusivity $d_{w}^{e}$, (c) intracellular diffusivity $d_{w}^{i}$, and (d) low-frequency mean conductivity *σ*_*L*_. The 3-rd and 6-th columns show the absolute values of difference between the corresponding results with 15 and 7 *b*-value DWIs, respectively.

In order to test the ability of the proposed method for more practical situations, we also used 7 and 4 *b*-value DWI data. In Fig 4, we compared the recovered extracellular volume fraction *α*, extracellular mean diffusivity $d_{w}^{e}$, intracellular diffusivity $d_{w}^{i}$, and low-frequency mean conductivity *σ*_*L*_ estimated with all *b*-value DWIs (15 *b*-value DWIs), 7 *b*-value DWIs (b = 300, 500, 700, 1000, 1400, 1800, 3600 s/mm^2^), and 4 *b*-value DWIs (b = 300, 1000, 1800, 3600 s/mm^2^) in the first imaging slice of the brain. The 4-th and 5-th columns in Fig 4 show the absolute differences between the corresponding results with 15 and 7 *b*-value DWIs and those with 15 and 4 *b*-value DWIs, respectively. Fig 5 shows the recovered results with 15 and 7 *b*-value DWIs using the three pool method. The differences of reconstructed results with respect to the number of *b*-values show that the proposed method stably recovers the low-frequency electrical properties with relatively small number of *b*-value DWIs. The averaged absolute differences (mean and standard deviation) between the corresponding results, *α*, $d_{w}^{e}$, $d_{w}^{i}$, and *σ*_*L*_, with 15 and 7 *b*-value DWIs were 0.03±0.04, 1.48±2.10 × 10^−4^mm^2^/s, 0.58±1.38 × 10^−4^mm^2^/s, and 0.04±0.06 S/m, respectively. The averaged absolute differences with 15 and 4 *b*-value DWIs were 0.05±0.06, 1.58±2.28 × 10^−4^mm^2^/s, 0.73±1.31 × 10^−4^mm^2^/s, and 0.05±0.07 S/m, respectively. On the other hand, in the three pool method, relatively bad values were observed (0.07±0.06, 7.46 ±7.16 × 10^−4^mm^2^/s, 1.00 ±1.58 × 10^−4^mm^2^/s, and 0.10±0.10 S/m, respectively).

We also used the Dice similarity coefficient (DSC) and the relative *L*^2^-error, as our comparison measures. For vectors **a** and **b**, DSC is defined as

$$\begin{array}{c} \text{DSC}=\frac{2\;|a\cdot b|}{{∥\;a\;∥}^{2}+{∥\;b\;∥}^{2}}. \end{array}$$ (15)

The relative *L*^2^-error is defined as

$$\begin{array}{c} L_{err}^{2}=\frac{∥\;a-b\;∥}{∥\;a\;∥}. \end{array}$$ (16)

For example, in the case of the extracellular volume fraction, **a** and **b** denote the extracellular volume fractions reconstructed with 15 *b*-values and sub-sampled *b*-values, respectively.

Tables 2 and 3 shows the DSC values and the relative *L*^2^-errors between the results with 15 *b*-value DWIs and sub-sampled *b*-value DWIs, respectively. For the proposed method compared to the three pool method, higher DSC values and lower relative *L*^2^-errors were reported, especially in the white matter region.

**Table 2:** DSC values between the results with 15 *b*-value DWIs and sub-sampled *b*-value DWIs in the first imaging slice of the brain.

	M*b*D-LF-CPI method				M*b*D-LF-CPI method				three pool method			
	(7 b-values)				(4 b-values)				(7 b-values)			
	Brain	CSF	GM	WM	Brain	CSF	GM	WM	Brain	CSF	GM	WM
*α*	0.994	0.999	0.992	0.991	0.990	0.998	0.987	0.985	0.958	0.987	0.894	0.893
$d_{w}^{e}$	0.979	0.999	0.976	0.949	0.977	0.999	0.976	0.942	0.912	0.993	0.905	0.884
$d_{w}^{i}$	0.990	0.830	0.999	0.998	0.990	0.862	0.997	0.995	0.976	0.732	0.993	0.993
*σ*_*L*_	0.990	0.999	0.979	0.972	0.986	0.999	0.970	0.965	0.960	0.994	0.938	0.873

**Table 3:** Relative *L*^2^-error between the results with 15 *b*-value DWIs and sub-sampled *b*-value DWIs in the first imaging slice of the brain.

	M*b*D-LF-CPI method				M*b*D-LF-CPI method				three pool method			
	(7 b-values)				(4 b-values)				(7 b-values)			
	Brain	CSF	GM	WM	Brain	CSF	GM	WM	Brain	CSF	GM	WM
*α*	0.036	0.023	0.057	0.058	0.075	0.043	0.099	0.107	0.187	0.112	0.342	0.318
$d_{w}^{e}$	0.075	0.023	0.118	0.152	0.090	0.038	0.121	0.212	0.221	0.109	0.315	0.290
$d_{w}^{i}$	0.064	0.411	0.025	0.034	0.062	0.375	0.047	0.058	0.099	0.562	0.072	0.059
*σ*_*L*_	0.055	0.012	0.134	0.116	0.094	0.034	0.227	0.171	0.171	0.094	0.257	0.353

Since there is no available data for the ratio of ion concentrations in ICS and ECS, we used the ratio *β* = 0.41 using some reference values [25]. To evaluate the influence of possible errors for *β*, we recovered the extracellular ion concentration ${\bar{c}}_{e}=\frac{\sigma_{H}}{\alpha d_{w}^{e}+\left(1-\alpha \right)\beta d_{w}^{i}}$ as *β* was increased from 0.31 to 0.56, respectively. For the true concentration ratio *β*_*t*_ and the assumed *β* = *β*_*t*_ + *β*_*ϵ*_, the extracellular concentration ${\bar{c}}_{e}$ can be expressed as

$$\begin{array}{c} \begin{array}{cc} {\bar{c}}_{e} & =\frac{\sigma_{H}}{\alpha d_{w}^{e}+\left(1-\alpha \right)\beta_{t}d_{w}^{i}}\frac{1}{1+\frac{\left(1-\alpha \right)\beta_{\epsilon }d_{w}^{i}}{\alpha d_{w}^{e}+\left(1-\alpha \right)\beta_{t}d_{w}^{i}}}\\ & ={\bar{c}}_{e}^{true}+O(\frac{\left(1-\alpha \right)\beta_{\epsilon }d_{w}^{i}}{\alpha d_{w}^{e}+\left(1-\alpha \right)\beta_{t}d_{w}^{i}}) \end{array} \end{array}$$ (17)

where ${\bar{c}}_{e}^{true}$ denotes the exact extracellular ion concentration and g(ϵ)=O(ϵ) means that the ratio $\frac{g(\epsilon )}{\epsilon }$ stays bounded as *ϵ* → 0. The relation (17) implies that the error between the estimated ${\bar{c}}_{e}$ and ${\bar{c}}_{e}^{true}$ mainly depends on extracellular volume fraction, extracellular mean diffusivity, and diffusion coefficient $d_{w}^{i}$.

**Fig 6:**
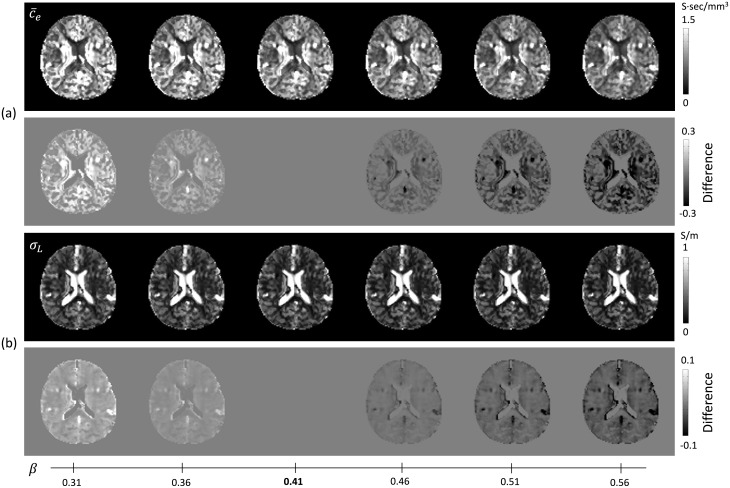
Reconstructed apparent extracellular ion concentration ${\bar{c}}_{e}$ and low-frequency mean conductivity *σ*_*L*_ as *β* changes. (a) Apparent extracellular ion concentration images by changing *β* value from 0.31 to 0.56 (first row) and the difference images of ${\bar{c}}_{e}\left(\beta \right)-{\bar{c}}_{e}\left(\beta =0.41\right)$ (second row), respectively. (b) Low-frequency conductivity images by changing *β* value from 0.31 to 0.56 (first row) and the difference images of *σ*_*L*_(*β*) − *σ*_*L*_(*β* = 0.41) (second row), respectively.

Fig 6 shows the reconstructed apparent extracellular ion concentration ${\bar{c}}_{e}$ and low-frequency mean conductivity *σ*_*L*_ as *β* changes. The second rows of (a) and (b) show the difference between the corresponding results with each *β* and *β* = 0.41, respectively. To compare the corresponding results, we calculated the relative ion concentration changes as

$$\begin{array}{c} Er\left(\beta \right)=\frac{|{\bar{c}}_{e}\left(\beta \right)-{\bar{c}}_{e}\left(\beta =0.41\right)|}{{\bar{c}}_{e}\left(\beta =0.41\right)}. \end{array}$$ (18)

The estimated relative ion concentration changes *Er*(*β*) in the brain region were 0.12, 0.06, 0, 0.05, 0.09, and 0.13 corresponding to *β* = 0.31, 0.36, 0.46, 0.51, and 0.56, respectively. As the ratio of ion concentration varies depending on the tissue structure and pathologic state, as shown in Fig 6, the estimated relative ion concentration changes *Er*(*β*) in the brain show that the recovered electrical properties were not significantly sensitive to the slightly changed *β* for the fixed *β* = 0.41.

**Fig 7:**
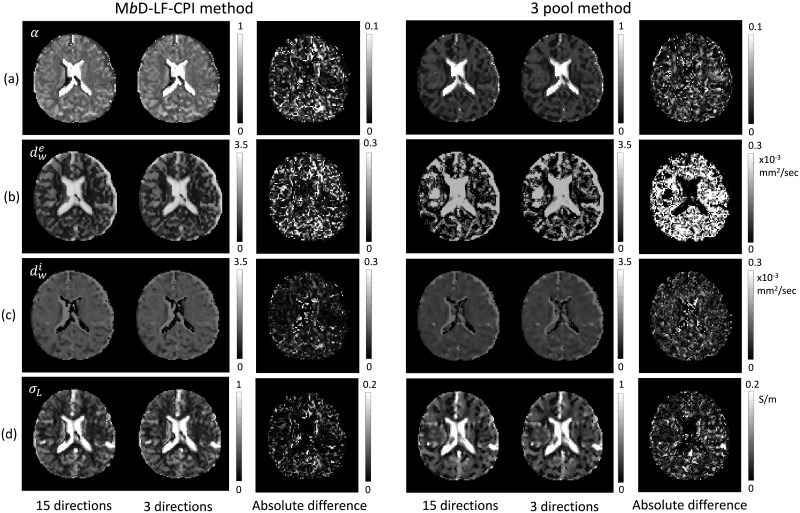
Comparison of reconstructed results estimated with 15 gradient directions and 3 orthogonal gradient directions in the first imaging slice of the brain. (a) extracellular volume fraction *α*, (b) extracellular mean diffusivity $d_{w}^{e}$, (c) intracellular diffusivity $d_{w}^{i}$, and (d) low-frequency mean conductivity *σ*_*L*_. The 3-rd and 6-th columns show the absolute values of difference between the corresponding results with 15 and 3 gradient directions, respectively.

In Fig 7, we compared the recovered extracellular volume fraction *α*, extracellular diffusivity $d_{w}^{e}$, intracellular diffusivity $d_{w}^{i}$, and low-frequency mean conductivity *σ*_*L*_ estimated with 15 gradient directions and 3 orthogonal gradient directions in the first imaging slice of the brain. The 3-rd and 6-th columns in Fig 7 show the absolute differences between the corresponding results with 15 and 3 gradient directions, respectively. The relative *L*^2^-errors of *α*, $d_{w}^{e}$, $d_{w}^{i}$, and *σ*_*L*_ with 15 and 3 gradient directions were 0.032, 0.048, 0.049, and 0.069, respectively. Here, the relative *L*^2^-errors were calculated using (16). **a** and **b** in (16) mean the values reconstructed with 15 gradient directions and 3 orthogonal gradient directions, respectively. For the three pool method, the corresponding relative *L*^2^-errors were 0.042, 0.072, 0.060, and 0.084.

## Discussion

To extract the low-frequency conductivity information from the high-frequency conductivity, we decomposed the recovered high-frequency conductivity by using MREPT technique from B1 phase measurements into a weighted combination of the conductivity of intracellular and extracellular compartments. The DWI for a fixed *b*-value assumes that all water molecules in a voxel have a single ADC, the ADC, however, reflects combined diffusion properties of intracellular and extracellular compartments. Multi-*b* value DWIs with multi-diffusion gradient directions have been widely studied to detect multi-diffusion coefficient and the intracellualr and extracellular volume fractions [23]. The water diffusion in ECS is less restricted and hindered than that in ICS. The *b*-value is a key parameter in the water diffusion and high *b*-values have been known to be more sensitive to ICS [32]. We found that the estimated low-frequency conductivity tensor using the proposed method was slightly low in the white matter region because the white matter was highly anisotropic due to the parallel orientation of the nerve fiber tracts.

To estimate the low-frequency electrical properties in ECS, we designed the multi-compartment model (7). The developed model estimated the microstructure of ICS reflected the averaged intracellular diffusion coefficient *d*_*ic*_ = 1.7 mm^2^/s and volume fraction *ν*_*ic*_, the extracellular diffusivity, and CSF compartment (diffusion coefficient *d*_*iso*_ = 3 mm^2^/s and volume fraction *ν*_*iso*_). The proposed method used several DWI signals for different *b*-values to recover the low-frequency conductivity in the brain region, the recovered volume fraction in ECS was, however, overestimated because the recovered extracellular microstructure still included unmyelinated neurons and other cells of the central nervous system. The hindered diffusion of free water molecules, related to the intrinsic diffusion coefficient of extracellular space and the tortuosity of the tissue, makes it difficult to distinguish the diffusion in the intracellular space, depending on the cell structures. To precisely estimate the low-frequency conductivity using only an MRI scanner, a new model for DWI to detect the diffusion-limited compartment and more exact estimation of microstructures in ECS are needed in the future work.

The proposed method recovers the ion concentration and the mean mobility in ECS using multi-*b*-value DWIs. In this paper, we focused on the reconstruction of the low-frequency conductivity tensor as a 3 × 3 positive definite symmetric matrix form. Due to the anisotropic conductivity tensor, the recovered low-frequency conductivity can be used to predict the current pathway and electric field distribution, which could be useful information for proving the therapeutic effects of electrical stimulation. Electrical brain stimulation (EBS) techniques are promising treatments for human disorders: transcranial direct current stimulation (tDCS), cranial electrotherapy stimulation (CES), electroconvulsive therapy (ECT), and deep brain stimulation (DBS), etc. EBS studies have relied on computational modeling using known reference conductivity values in the brain and thus the specificity of each individual’s brain is an obstacle of EBS. The proposed method can provide a way to investigate the EBS without real experiments.

DTI is currently a widely used technique for visualizing major fiber orientation of the brain in fitting a Gaussian distribution of diffusion, but DTI also has limitations in characterizing the diffusion process in areas of low anisotropy and complex fiber structure in a voxel. Using a higher number of diffusion gradient directions, various techniques have been introduced to overcome the limitation of DTI [5]. Although high angular methods use more gradient encoding directions and high gradient strength (*b*-value) than DTI, we believe that anisotropic electrical properties also can be extended to characterize the complex anisotropy depending on the fiber structures in a voxel.

We expect that the recovered concentration image can be applied to the relevant diseases. There are many diseases related to ion channels: physiological disorders (myotonias, Brugada syndrome, malignant hyperthermia, myasthenia), neuronal disorders (epilepsy and episodic ataxia, retinal diseases, Alzheimer’s disease, Parkinson’s disease, schizpphrenia), kidney disorders (policystic kidney disease, hyperinsulinemic hypoglycemia of infancy and cystic fibrosis, congenital stationary night blindness), etc. [33, 34]. Hence, rigorous analysis of the ion concentration characteristics by using a noninvasive imaging modality such as MRI is crucial to the pathophysiolgy of neurodegenerative processes.

## Conclusion

The water diffusion weighted imaging (DWI) measures the random Brownian motion of water molecules within a voxel, which related to the mobility of the water molecules by the Einstein relation. We investigated the decomposed high-frequency conductivity as the apparent total ion concentration and the mobility terms. MREPT uses the B1 mapping technique to provide the high-frequency conductivity distribution that reflects the intracellular and extracellular effects at the Larmor frequency of an MRI scanner. By measuring DWI data for multi-*b*-value and the recovered high-frequency conductivity distribution, we proposed a new multi-compartment model to estimate the extracellular volume fraction, the extracellular mean diffusivity, and the volume fraction of CSF. Using the estimated parameters, the high-frequency conductivity is decomposed into the apparent ion concentration and the extracellular mean mobility. A human experiment verified that the proposed method has the potential to rapidly recover the low-frequency electrical properties without any additional external injection current.

## Supporting information

S1 FigRecovered results.(a) Recovered high-frequency conductivity *σ*_*H*_. (b) Recovered extracellular volume fraction *α*, extracellular mean diffusivity $d_{w}^{e}$, intracellular diffusivity $d_{w}^{i}$, the apparent extracellular ion concentration ${\bar{c}}_{e}$ and low-frequency mean conductivity *σ*_*L*_ images, respectively, using M*b*D-LF-CPI method. (c) Recovered results corresponding to (b) using the three pool model method.(EPS)

S2 Fig Diffusion and conductivity tensors. (a) Water molecule diffusion tensor using the *b* value of 1000 s/mm^2^ in the first slice. (b) Reconstructed low-frequency conductivity tensor images using M*b*D-LF-CPI method. (c) Reconstructed low-frequency conductivity tensor images using the three pool model.
(EPS)

S1 TableResults of the proposed M*b*D-LF-CPI method and the three pool method.Estimated high-frequency conductivity *σ*_*H*_, extracellular ion concentration ${\bar{c}}_{e}$, low-frequency mean conductivity *σ*_*L*_, diagonal components of reconstructed low-frequency conductivity tensor **C**_*L*_ measured within the ROIs.(PDF)
