# Supplementary material for: Extracellular electrical conductivity property imaging by decomposition of high-frequency conductivity at Larmor-frequency using multi-b-value diffusion-weighted imaging
Source: PLoS One. 2020 Apr 8;15(4):e0230903. doi: 10.1371/journal.pone.0230903 (PMC7141654; doi:10.1371/journal.pone.0230903)
Supplement: S1 Table — Estimated high-frequency conductivity σH, extracellular ion concentration c¯e, low-frequency mean conductivity σL, diagonal components of reconstructed low-frequency conductivity tensor CL measured within the ROIs. (PDF) [file pone.0230903.s003.pdf]

(a) M*b*D-LF-CPI method

	σ_H	$\bar{c}_{ec}$	σ_L	C_{11}	C_{22}	C_{33}	Pixels
CSF	1.17 ±0.59	0.46 ±0.27	1.23 ±0.72	1.12 ±0.64	1.28 ±0.76	1.30 ±0.80	267
GM	0.55 ±0.18	0.97 ±0.49	0.30 ±0.16	0.32 ±0.17	0.30 ±0.16	0.29 ±0.16	1851
WM	0.46 ±0.15	0.97 ±0.38	0.22 ±0.13	0.22 ±0.14	0.21 ±0.13	0.24 ±0.15	1756

(b) 3 pool method

	σ_H	$\bar{c}_{ec}$	σ_L	C_{11}	C_{22}	C_{33}	Pixels
CSF	1.13 ±0.60	0.45 ±0.27	1.23 ±0.72	1.12 ±0.64	1.27 ±0.76	1.30 ±0.80	267
GM	0.55 ±0.18	0.86 ±0.46	0.33 ±0.16	0.35 ±0.19	0.33 ±0.17	0.32 ±0.17	1851
WM	0.46 ±0.15	0.71 ±0.34	0.30 ±0.12	0.29 ±0.13	0.28 ±0.13	0.32 ±0.15	1756
